# Perceptions of Scholars in the Field of Economics on Co-Authorship Associations: Evidence from an International Survey

**DOI:** 10.1371/journal.pone.0157633

**Published:** 2016-06-20

**Authors:** Sameer Kumar, Kuru Ratnavelu

**Affiliations:** 1 Asia-Europe Institute, University of Malaya, Kuala Lumpur, 50603, Malaysia; 2 Institute of Mathematical Studies, Faculty of Science, University of Malaya, Kuala Lumpur, 50603, Malaysia; Universidad de Alicante, ITALY

## Abstract

Scholars (*n* = 580) from 69 countries who had contributed articles in the field of Economics during the year 2015 participated in a survey that gauged their perceptions of various aspects of co-authorship, including its benefits, motivations, working relationships, order of authorship and association preferences. Among the main findings, significant differences emerged in the proportion of co-authored papers based on age, gender and number of years the researchers had spent in their present institution. Female scholars had a greater proportion of co-authored papers than male scholars. Respondents considered improved quality of paper, contribution of mutual expertise, and division of labor as the biggest benefits of and motivation for co-authorship. Contrary to common perceptions that Economics researchers used a predominantly alphabetical order of authorship, our study found that a considerable percentage of respondents (34.5%) had practiced an order of authorship based on the significance of the authors’ contribution to the work. The relative importance of tasks differed significantly according to whether researchers co-authored as mentors or co-authored as colleagues. Lastly, researchers were found to associate, to varying degrees, with other researchers based on socio-academic parameters, such as nationality, ethnicity, gender, professional position and friendship. The study indicates that Economics authors perceive co-authorship as a rewarding endeavor. Nonetheless, the level of contribution and even the choice of association itself as a co-author depends to a great extent on the type of working relationship and socio-academic factors.

## Introduction

In recent years, a large body of literature has used secondary data obtained from international databases to understand co-authorship behavior among scholars. In contrast, comparatively fewer studies have directly assessed scholars’ perceptions of co-authorship associations. Using an online questionnaire, we surveyed researchers in the field of Economics on four aspects of co-authorship: (1) benefits and motivations of co-authorship; (2) sharing of work when writing papers in relation to two distinct working relationships, that of a mentor and of a colleague; (3) order of authorship; and (4) preference of association with co-authors based on socio- academic factors. The results of the survey are presented in this study.

Co-authorship in research articles, considered a reliable proxy for research collaboration, has been extensively investigated [1–3]. Scientists communicate with one another to exchange opinions, share research results and write research papers [4]. On the one hand, communication among scientists could start with a simple discussion that leads to collaboration on a research project. On the other hand, scientists may decide to collaborate with scientists with whom they are already acquainted, knowing well their ability to carry out a particular research project. In another scenario, prospective collaborators can meet at conferences or at other forums and form an "invisible college" [5]. These informal exchanges may lead scholars to find a shared interest in a topic and to make a decision to collaborate on a research paper. Hence, various reasons could bring a group of researchers together.

Collaboration has several benefits. Katz [6], for example, mentioned factors that promote collaboration, including funding patterns; scientific popularity, visibility and recognition; the rationalization of scientific manpower; the demands of complex large-scale instrumentation; increasing specialization in science; the degree of advancement of a particular discipline; the professionalization of science; the need to gain experience and train researchers; the desire to increase cross-fertilization of ideas and techniques; and decreases in spatial distance. However, Katz [6] also stated that these factors, which are derived from the literature, are far from complete, as research collaboration is a social process and researchers have reasons to collaborate just as people have reasons to communicate.

At the same time, collaboration may have certain disadvantages, as it requires extra time to coordinate with all the stakeholders involved in a project and the coordination of especially large multi-institutional collaboration can be costly [7]. Apart from this, the problems of assigning credit to the authors may dissuade some, as they may not feel ‘recognized’. Research credit is an important currency in the career of researchers, and not being given due credit would reduce accountability, which often slows down research progress and lowers the quality of research findings [8, 9]. Moreover, unethical practices, such as conducting clinical practices that may be banned in some countries but not prohibited in other countries, is another negative aspect of research collaboration [10]. Collaboration is a key mechanism for mentoring graduate students and post-doctoral researchers. Pressure to publish [11] for promotion and/or tenure or to fulfil the publication requirements to remain in one’s job are strong motivations for collaboration.

Due to the availability of quality bibliometric data from sources such as Scopus and Web of Science, there has been a trend among Information Science researchers towards carrying out studies using secondary data. New insights into the topologies of networks have encouraged researchers to also look at co-authorship from the perspective of networks [12], and this has contributed to the emergence of a new set of bibliometric studies. Co-authorship effects on research productivity [13], centrality measures and their effect on research performance, the formation of research communities and research landscapes are a few examples of studies commonly performed using bibliometric data [14–19]. However, comparatively fewer studies have used primary data to gauge researchers’ perceptions of co-authorship, and even fewer studies addressed this topic from the point of view of academic economists. Among the few examples are a questionnaire survey by Hart [20], who examined the attitudes and behaviors of 98 academic librarians and reported the main reasons for their collaboration, including the author-order protocols followed, among others. Additionally, Melin [21] collected responses from 195 scholars to investigate the effects of collaboration at the individual level.

The present study attempts to gauge the perceptions of Economics authors on co-authorship associations. The fact that the survey is worldwide, is recent and includes a diverse set of questions makes the study and results more interesting. In contrast to previous empirical studies (i.e., [20, 21]), our study addresses a diverse set of research questions, which includes not only the perceived benefits and motivations of co-authorship and author-order protocols but also their preferences in associating with other researchers based on socio-academic parameters.

The survey was administered to researchers who had contributed to the field of Economics in 2015 through publications in journals indexed in Thomson Reuters SSCI databases. Economics is one of the foremost fields in the Social Sciences that has remained in tandem with the growth of the entire discipline of Social Science [22]. Unsurprisingly, Economics has remained the subject of several bibliometric studies [22–25]. The respondents in our survey were asked pressing questions related to co-authorship, i.e., what is the percentage of papers co-authored by the researchers in their lifetime? What do the researchers feel or perceive are the benefits of co-authorship (i.e., sharing of expertise or increase in number of publications for promotion, rewards, etc.)? What is the general order of authorship based on, significant contribution to work or alphabetical order? Traditionally, Economics papers have been known to follow the alphabetical order of authorship [26]. Hence, it would be interesting to see whether this really is the case with the researchers from our sample. We also wanted to know whether there is a difference in the contribution of researchers according to whether they are working as a mentor or working with a colleague while carrying out the various tasks associated with completing a study (i.e., writing paper or designing study). One of the least researched aspects of co-authorship involves the understanding of whether authors prefer associating with others for specific reasons, such as gender, nationality or professional position, among others. Thus, we also asked our respondents about these factors.

Specifically, we formulated the following questions:

1. Do authors prefer co-authorship to solo paper writing?
2. Is there any significant difference in the proportion of co-authored papers based on demographic and other parameters, such as age, gender, number of years in the present institution, etc.?
3. What are the perceived benefits of and motivations for co-authorship?
4. What is the practiced protocol of author-order based on, significant contribution to the work or alphabetical order?
5. In producing a research paper, is there a significant difference regarding the importance of tasks according to whether the researcher is a mentor or a colleague?
6. Do authors associate with others based on specific socio-academic parameters, such as race, gender, nationality, professional position or field of research?

The findings of the study provide insights into co-authorship associations from the direct experience of researchers.

In the next section, we discuss the research methods. We then discuss the results and, finally, end with our concluding thoughts.

## Materials and Methods

Ethics Statement: The University of Malaya Medical Centre–REC [27] states that “Researches that may not require ethical review by the MEC are (a) research solely involving the use of educational tests (cognitive, diagnostic, aptitude, achievement), survey procedures, interview procedures and data collection in the public domain, diagnostic and therapeutic procedures that are an accepted part of treatment and are recognized as a current practice by the appropriate professional body”. The data were collected using an anonymous online survey, and no direct human interaction was involved. Those interested in participating were informed that participation in the online survey is voluntary; they could also discontinue participation in the study at any time. Hence the very completion and submission of online form is sufficient evidence of consent.

Authors’ names and their e-mail IDs were extracted from ‘Articles’ indexed in the year 2015 in the Web of Science with ‘Economics’ as the subject category. A total of 1043 email addresses were extracted from the records using simple random sampling. A web-based questionnaire in the form of a google survey was posted online, and an individualized email was then sent to all 1043 researchers requesting them to participate in the online survey. The questionnaire assessed the respondents’ demographic characteristics (age, nationality, name of institution, qualification or professional position, etc.) and their perceptions of four aspects of co-authorship: benefits of co-authorship, models of working relationships, author order and working preferences. In the last section, respondents were asked to write about any other issue or provide additional information about co-authorship.

The first set of emails (as a pilot test) was sent to approximately 100 respondents. Based on the average of the first 50 responses, the relevant questionnaire sub-sets had acceptable internal reliability, as indicated by a Cronbach’s alpha of 0.809. A request to respond to the survey was then sent to the rest of the e-mail IDs. Overall, 114 emails either bounced or returned with an automated vacation message.

**Fig 1:**
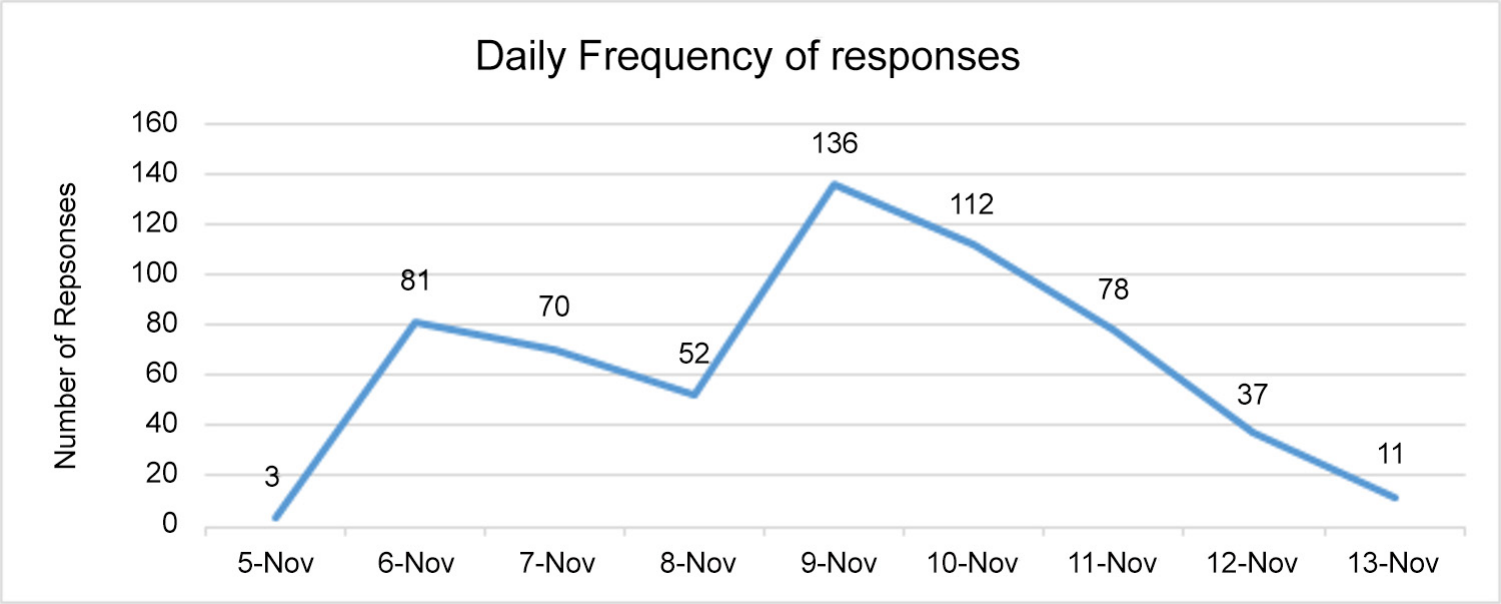
Frequency of responses received.

Between November 5^th^ and November 12^th^, 2015, we received 581 responses (see Fig 1), reflecting an overall response rate of approximately 62.54%. One of the records had to be discarded due to the absence of demographic data, bringing the total number of valid records to 580.

To design the questionnaire, Hart [20] was used as a guide. The analyses were determined based on our research questions. For example, three sections–benefits and motivations of co-authorship, order of authorship and work preference—were assessed mainly by descriptive analysis, whereas the mentor-colleague working relationship was examined using inferential statistics.

The respondents (*n* = 580) represented 69 countries (see Tables 1 and 2). As can be observed, over two-thirds of responses came from Europe and the US. These respondents had their primary place of work in these countries at the time of responding. We also noticed that close to 23% of the respondents did not work in their home countries or had dual nationality; thus, they were either working abroad or were immigrants.

**Table 1:** Frequency distribution of respondents as per country of work.

Country	Freq	%	Country	Freq	%	Country	Freq	%
Argentina	2	.3	Hong Kong	1	.2	Poland	6	1.0
Australia	25	4.3	Hungary	2	.3	Portugal	7	1.2
Austria	7	1.2	India	12	2.1	Romania	5	.9
Belgium	9	1.6	Indonesia	1	.2	Russia	7	1.2
Brazil	7	1.2	Iran	2	.3	Saudi Arabia	2	.3
Bulgaria	2	.3	Ireland	3	.5	Serbia	2	.3
Cameroon	1	.2	Israel	5	.9	Singapore	5	.9
Canada	19	3.3	Italy	59	10.2	Slovakia	1	.2
Chile	2	.3	Japan	13	2.2	Slovenia	2	.3
China	2	.3	Kenya	1	.2	South Africa	1	.2
Colombia	1	.2	Lebanon	1	.2	South Korea	4	.7
Croatia	3	.5	Lithuania	2	.3	Spain	28	4.8
Cyprus	1	.2	Luxembourg	2	.3	Sweden	8	1.4
Czech Republic	6	1.0	Macedonia	1	.2	Switzerland	11	1.9
Denmark	6	1.0	Malawi	1	.2	Taiwan	2	.3
Ecuador	1	.2	Malaysia	9	1.6	Thailand	1	.2
Egypt	1	.2	Mexico	5	.9	Tunisia	1	.2
Estonia	1	.2	Netherlands	15	2.6	Turkey	4	.7
Finland	8	1.4	New Zealand	5	.9	UAE	3	.5
France	27	4.7	Nigeria	2	.3	United Kingdom	35	6.0
Germany	27	4.7	Norway	8	1.4	Uruguay	3	.5
Ghana	1	.2	Pakistan	1	.2	USA	117	20.2
Greece	10	1.7	Peru	2	.3	Vietnam	3	.5
						**Overall Total**	**580 **	**100.0 **

**Table 2:** Frequency distribution of respondents as per continent of work.

Continent	Frequency	%
Oceania	30	5.2
Asia	78	13.4
Africa	9	1.6
Europe	304	52.4
South America	18	3.1
North America	141	24.3
**Total**	**580**	**100.0**

Most respondents worked in academia, held a PhD, and were above 35 years of age and married (see Table 3). The male to female ratio of the sample was 3:1, meaning there were three male respondents to each female respondent. This also largely represents the overall current picture of academia, which has a higher ratio of males [28, 29]. More than half of the respondents have been working in their present institution for 6 or more years.

**Table 3:** Characteristics of respondents.

Descriptives	Valid (n)		Frequency	Valid Percent
**Gender**	580	Male	447	74.8
		Female	133	25.2
**Age**	580	less than 35 years	114	19.7
		35–45	251	43.3
		46–55	118	20.3
		56 and above	97	16.7
**Marital Status**	580	Single	103	17.8
		Married	449	77.4
		Other	28	4.8
**Highest Qualification**	580	PhD	541	93.3
		Masters	27	4.7
		Other	12	2.1
**Institution of work**	577	College	9	1.6
		University	477	82.2
		Research Institute	53	9.1
		Other	38	6.6
**Years of service at**	580	Less than 1 year	34	5.9
**current institution**		1–5 years	208	35.9
		6–10 years	127	21.9
		More than 10 years	211	36.4
**Professional Position**	569	Lecturer	25	4.3
		Senior Lecturer	32	5.5
		Assistant Professor	103	17.8
		Associate Professor	112	19.3
		Professor	190	32.8
		Post Doc	13	2.2
		Student	10	1.7
		Economist (not holding academic position)	16	2.8
		Researcher/Scientist	42	7.2
		Other	26	4.5

## Results and Discussion

### Percentage of papers co-authored by researchers during their academic career

The incidence of co-authorship in Economics rose sharply in the 1970s [30]. Increasing specialization, changes in institutional incentives for publication, along with a host of other reasons, have brought about a marked trend toward co-authored articles. The trend towards co-authorship was perhaps ‘one of the most violent transitions that can be measured in recent trends of scientific manpower and literature’ (p. 89) [31].

In our study, we asked the researchers about the percentage of papers, out of the total number of papers authored by them, they had co-authored. Overall, 99% of all respondents had co-authored at least some portion of their papers during their career. Approximately 75% of the respondents said that they had co-authored two-thirds or more of their papers, and over 50% mentioned that they had co-authored all or almost all of their papers (see Table 4). These figures decidedly show that co-authoring a paper is almost a norm in the Economics academic community. A number of other studies have reported a similar trend in the rise of multiple authored papers in every scientific discipline within and across countries [10]. Large industrial projects, improvements in communication facilities led by information technology, and the mobility of researchers have created a fertile ground for researchers to work in groups [31, 32]. Economics, an important social science discipline, has also followed this trend, as is evident from our results.

**Table 4:** Frequency of respondents’ % of papers co-authored.

Proportion of co-authored papers	Freq	%
None (All have been solo written)	6	1.0
Very few	42	7.2
About one-third	39	6.7
About half	52	9.0
About two-thirds	138	23.8
Almost all papers	213	36.7
All papers	90	15.5
**Total**	**580**	**100.0**

We next examined any significant difference in the proportion of co-authored papers based on age, gender, marital status, institution type, professional experience, and position or qualification. A Kruskal-Wallis test and median test (both are K-independent samples non-parametric tests) were conducted to assess significant differences in the proportion of co-authored papers based on demographic variables (see Table 5). A significant difference in the proportion of co-authored works was observed between males and female researchers (asymp. sig. 2 tailed = 0.01). Female researchers seemed to have co-authored a greater number of works compared to their male counterparts. Female authors also tended to have a greater number of collaborators. A study by Bozeman and Gaughan [33] found that women actually have more collaborators on average compared to male researchers.

**Table 5:** Statistical test to determine significant difference in the proportion of co-authored papers based on demographic profile.

	Kruskal-Wallis Test			Median Test		
Variable	Chi-square	df	Asymp. Sig.	Chi-square	df	Asymp. Sig.
Age	51.647	3	.000*	49.767	3	.000*
Gender	11.067	1	0.01^+^	5.845	1	.016^+^
Marital Status	2.949	2	0.229	9.222	2	0.10
No. of years of service in current institution	14.201	3	0.003*	23.777	3	.000*
Continent	8.147	5	0.148	10.752	5	0.57

*significant at p<0.01

^+^significant at p<0.05

A significant difference was observed in terms of age. Researchers who were 56 years old and above co-authored significantly less articles compared to researchers 45 years old and below. Older authors tended to have a different research styles compared to their younger counterparts [30]. It is likely that researchers older than 56 years of age published some of their early career papers without co-authors or had a different research style. Differences in the types of skills and interpersonal relationships between older and younger researchers may also be responsible for their dissimilar co-authorship patterns [34]. Again, a significant difference was observed in the number of years spent in present institution and proportion of co-authored papers. Those who had spent more than 10 years in their current institution had a smaller proportion of co-authored papers compared to those who had worked in their current institution for fewer numbers of years. Those who had just joined the institution (<1 year) had the highest proportion of co-authored papers. The results give credence to the fact that co-authorship in research papers is a phenomenon that has become more prevalent in recent years, and young researchers or those who have recently joined a university or academic institution recognize its inevitability.

### Benefits and Motivations of Co-authorship

Researchers collaborate for several reasons. The primary basis for research collaboration is that it brings individuals together to work on a project (i.e., research study) that could not be completed by a single author. Therefore, bringing together multiple talents is the hallmark of research collaboration. Theoretically, this is true, but in cases of honorary authorship and ghost authorship, co-authorship may not be an actual reflection of research collaboration.

We asked the respondents to rate the potential benefits of or motivation for collaboration on a 4-point scale (least being ‘not important’ and highest being ‘most important’; with a given weight of 0 to 3, respectively). The benefits are presented in descending order of importance, in Table 6.

**Table 6:** Motivations and Benefits of research collaboration.

Benefits and motivations	Valid *N*	Mean	Std. Deviation
Improvement in the quality of research paper	580	2.43	.678
Mutual gain of expertise among co-authors	580	2.35	.733
Division of labor	580	1.95	.895
Opportunity to work with co-authors from International institutions	580	1.61	1.003
Establishing further networks	580	1.52	.972
Increase in the no. of publications thereby helping in promotion or tenure	580	1.49	1.003
Mentor a junior colleague	580	1.48	.954
Opportunity to work on multi-disciplinary areas	580	1.46	1.031
Be mentored by a senior colleague	580	1.40	1.043
Opportunity to get to know a colleague	580	1.32	.931
Opportunity to be part of a large international or cross-country research	580	1.28	1.023
Reduction in research costs because of sharing of resources	580	.96	.978

Beaver [35] cited 18 potential reasons for why researchers collaborate, including access to expertise, sharing of resources, improved access to funds, professional advancement, learning tacit knowledge, progressing more rapidly, tackling larger or bigger problems, enhancing productivity, getting to know people, learning new skills, satisfying curiosity, sharing the excitement of an area with other people, reducing errors, staying focused on research, reducing isolation, advancing education (i.e., student education), advancing knowledge, but also having fun. With these 18 reasons, Beaver practically summarized a large body of literature that has examined reasons for why researchers collaborate.

Our study found that the most important reason for collaboration is that it improves the quality of the paper. Improvement in paper quality is also likely to increase the chances of acceptance in a journal. The improved quality of a paper is paramount during a journal peer-review process. Presser [36] found that multiple-authored papers were more likely to be accepted for publication compared to single-authored articles. In his studies, he noted that PhD departments (i.e., department with a PhD program) received more favorable reviews compared to non-PhD departments. Citing a case, Presser [36] also showed that individual papers written by a PhD department had a 76.7% rejection rate compared to 60% rejection rate for two-author papers. The decrease in rejection rate for multiple-authored papers supports the notion that quality improvement does occur when authors co-author a paper. Beaver and Rosen [37] investigated papers based on journal prestige and found that quality journals contained a greater number of multi-authored articles. When Melin [21] asked scholars about the main benefit of collaboration, 68% indicated increased knowledge and high scientific quality of papers produced. However, the link between collaboration and quality is often debated. For example, in a study involving two important journals of Academic Librarianship, Hart [38] found no evidence to suggest that co-authorship resulted in better quality articles.

The second most important reason for research collaboration, consistent with our study, is ‘mutual gain of expertise among co-authors’. Collaboration increases scientific credibility, as researchers get a chance to work with other researchers from diverse fields and backgrounds, producing a greater number of works of better quality [10, 39]. When asked about the major reason for collaboration, over 60% of respondents in the study by Melin [21] reported co-authors’ special competence and co-authors’ availability of special data and equipment.

Division of labor [21], where authors are in a position to divide their work among themselves, has been cited in our study as the third most important reason why authors collaborate. Division of labor can be very fruitful. For example, if three authors collaborate on a paper, one can focus on the literature review, the other on research design, and yet another on data analyses.

In this regard, a respondent commented:

‘It improves the efficiency of producing a paper and helps produce a better paper, as the work load is shared and each team member focuses on the areas of their strength’

Research collaboration enables the sharing of expertise and exchange of ideas [4, 21]. As more than one person is looking into the quality, accuracy, and meaning of the results, it increases scientific reliability and the probability of success.

Another respondent comments:

‘Complementarity of skills and knowledge between co-authors is the most important decision in choosing collaborators’

‘Opportunity to work with co-authors from international institutions’ and ‘Establishing further networks’ were mentioned as the 4^th^ and 5^th^ top benefits and motivations of research collaboration, respectively. Internationality is fast becoming an essential criterion for research collaborations. A good number of recent studies have shown that international articles are being cited twice as much as locally co-authored papers [40]. Mark Granovetter’s [41] “strength of weak ties” refers to the idea of innovation coming from “outside” (international ties) as opposed to the “strong ties” (local ties) in which authors are situated in. Internationally collaborated projects also tend to provide a diverse and innovative perspective, which might be missed if researchers collaborate only with their local team members. Researchers’ look forward to expanding their research network, as it is good for their research and for establishing their prominence in the research community. This notion takes further strength from the idea of transitivity, a common term in social networks literature. ‘Transitivity’ hypothesizes that if researcher A is connected to researcher B and researcher B is connected to researcher C, there is also high likelihood that eventually researcher A will associate with researcher C. Hence, it is not surprising that “establishing further networks” is an important benefit or motivation. A significant gender difference was observed in perceiving “establishing further networks” as a benefit (Asymp. Sig. 2-tailed = 0.001). Female authors seemed to assign more importance to this benefit compared to their male counterparts. Coupled with the fact that, in our sample, women researchers co-authored more papers compared to male researchers, the finding further suggests that female researchers may indeed be more social, looking for more collaborations compared to male researchers. A study by [33] also indicated that women researchers tend to have, on an average, more collaborators compared to their male counterparts.

A Kruskal-Wallis Test showed significant difference in the choice of benefits and motivations with respect to the age of researchers. While ‘Increase in the no. of publications to obtain promotion or tenure’ was not a motivating factor for researchers older than 56 years, it was one of the most important factors for those younger than 35 years of age. Similarly, ‘being mentored by senior colleague’ was a more important factor for younger rather than older researchers, while ‘mentoring a junior colleague’ was a more important factor for older colleagues than for younger ones.

A significant difference (Asymp. Sig. 2-tailed = 0.000) was again observed between researchers residing in different regions of the world. In contrast to researchers in North America (mainly the US), researchers in Asia or Africa consider working with international institutions as an important benefit or motivation. Similarly, North American scientists gave comparatively less importance to ‘Establishing further networks’ compared to researchers in Africa or South America.

The respondents considered that co-authorship could potentially increase the total number of publications of a researcher (6^th^ top benefit and motivation for co-authorship). One of the most consistent findings in the literature has been the high degree of correlation between collaboration and research productivity [4, 42]. Zuckerman [43] interviewed 41 Nobel Prize winners and identified a strong relationship between collaboration and productivity. Indeed, Nobel laureates were more apt to collaborate compared to a matched sample of scientists. However, owing to strains resulting from prestige, collaboration ties (with most of these terminating) decreased soon after the award. Pao [44] noted that musicologists who collaborated the most were also the most productive. The increase in the number of publications increases the prestige of researchers in the research community. As the influence of researchers grows, other researchers show their interest in working with them, further increasing the number of publications. Collaboration has a cumulative effect that increases the popularity of the researcher.

Landry, Traore [45] carried out an econometric analysis and showed that collaboration within universities, industries, or institutions may indeed increase academic productivity. However, productivity may vary according to the geographical closeness of the partners and their field of research. The researcher also found that collaboration between universities and industry was far more productive compared to collaborations between universities and universities and other institutions. Lee and Bozeman [46] conducted one of the most significant studies on the effect of collaboration and scientific productivity. They examined 443 academic scientists affiliated with university research centers in the US and found that the net effect of collaboration on research productivity was less clear. The researchers conducted a ‘fractional count’ by dividing the number of publications by number of authors and found that number of collaborators was not a significant predictor of productivity. However, they also concurred that their findings were conducted at an individual level while the major benefits of collaboration may accrue to groups, institutions and research fields.

Research collaborations could also benefit researchers across different nations. A respondent’s comment below gives a fair impression of how a researcher from one nation could benefit from aligning with a researcher from another nation.

“When I am writing a paper that compares economic outcomes in the USA with those in another country or I am working on a paper about a country other than the USA, I very much prefer to work with a researcher from that country.”

Another respondent from the US noted:

“I have performed a few survey studies in China, and having Chinese scholars involved as co-authors was critically important to have access to survey respondents. I assume this may be the case with many studies involving respondents in other countries”

Informal and formal collaboration could bring about international co-operation even when relations between countries are strained [47]. It could also heal post-war wounds by facilitating the redirection of military research funds to peace-time applications [10]. Scientific collaboration also has several socio-economic benefits. It could spread the financial risk of research for businesses over the long term. By collaborating with developing countries, companies can hire scientists from developing countries at much lower rates compared to those prevalent in advanced countries [10].

Our findings are in line with the empirical study conducted by Hart [20], who analyzed the responses from the authors of multiple-authored articles published in two journals on academic librarianship and found that, among the nine potential benefits, improved quality of the article, co-authors’ expertise, valuable ideas received from the co-author and division of labor were among the most important reasons for collaboration.

### Authorship Order

First authorship is often considered significant in multiple-authored papers, a practice that reflects research collaboration. It is widely recognized that the first author provides a major contribution to the paper. In some disciplines, the author order is based on the alphabetical sorting of surnames; however, first authorship is considered important in most disciplines. Some landmark studies are known by their first author, lending support to the impression that by being the first author, he or she plays a pivotal role in a particular research [48]. In essence, the order of authoring is an adaptive device, which symbolizes authors’ relative contribution to research [49].

We asked the respondents about how their names generally appear on research papers they have co-authored. Three options were given: in order of significant contribution; alphabetically—indicating an equal contribution by each author; and alphabetically—with no intent to indicate significant contribution. Respondents had to choose from 7 options. The results are provided in Table 7.

**Table 7:** Order of authorship.

Portion of papers	In order of significant Contribution		Alphabetically, indicating an equal contribution by each author		Alphabetically, with no intent to indicate significant contribution	
	Frequency	Percent	Frequency	Percent	Frequency	Percent
In none of my papers	152	26.2	227	39.1	267	46.0
In very few of my papers	146	25.2	88	15.2	76	13.1
In about one-third of my papers	45	7.8	32	5.5	26	4.5
In about half of my papers	37	6.4	33	5.7	28	4.8
In about two-thirds of my papers	27	4.7	39	6.7	24	4.1
In almost all my papers	84	14.5	85	14.7	87	15.0
In all my papers	89	15.3	76	13.1	72	12.4
Total	580	100.0	580	100.0	580	100.0
**Mean Score**		**2.4**		**2.2**		**2.0**

The field of Economics is known for following the alphabetical order of authorship [26, 50]. From our results, however, no clear trend emerged in this direction (see Table 6). On the one hand, 343 (59.1%) respondents mentioned that they had either never practiced author-order based on significant contribution or had authored only one-third or less of their papers this way. On the other hand, approximately 34.5% of respondents authored their papers in the order of significant contribution (from two-thirds of their papers to all of their papers).

Authorship order has been changing over time. Drenth [51] carried out a study to assess the change in the number and profile of authors who had contributed articles to the BMJ (previously called the ‘British Medical Journal’, now only referred to as ‘the BMJ’) over a 20-year period and found a shift in the hierarchical order of authorship over time, with senior authors (professors and chairpersons) moving to the first authorship at the cost of other contributors, such as consultants and lecturers.

Is the trend in Economics changing, too? It is difficult to conclude from the data. Although a slight shift can be observed towards alphabetical listing, a sizable percentage also had either all papers or almost all papers in the order of significant contribution.

Fine and Kurdek [52] cited American Psychological Association’s (APA) ethics committee’s policy on authorship of articles based on dissertations to determine authorship credit and the authorship order of faculty–student collaboration. The policy statement indicates that dissertation supervisors must be included as authors in such articles only if they have provided ‘significant contributions’ to the study. In such situations, only second authorship is appropriate for supervisors, as a dissertation is an original study by the student; thus, first authorship is always reserved for the student.

As a respondent noted:

In our institution […], in order for a PhD student to graduate with the PhD degree, they must publish a paper in an SSCI journal. This means that the supervisor must work very closely and mentor the student. For that reason, I always put the student's name first. Otherwise, the order of the authors is usually in alphabetical order unless my colleague clearly makes a larger contribution. In these cases, their name is placed first in the publication. That is the case in the paper you cited in your e-mail. The authors are put in the order of the number of hours they spent on it.

Although The International Committee of Medical Journal Editors (ICJME) also has specific criteria when dealing with authorship issues [53], honorary authorship (where the author becomes part of the author list without providing significant contribution), is still a major issue. As some respondents expressed:

“I did not benefit much from joint works in the past, as I had to do almost all aspects of research and publication. Unlike the past, I now work with my PhD students, who do the research work, and I help to generate ideas, share models and techniques, improve writing, undertake editing, publishing and so on. Now I like to work with others who, in my view, are not 'free riders' but are prepared to spend time and to share analytical skills where I have weaknesses to raise the quality of my papers. Most of my publications are sole authored; my future joint works should be genuinely collaborative”.

“Based on what I see in the literature, it seems that for junior academics, co-authorship with senior academics is a way to get published in higher ranking journals. Additionally, what is even more common is that you see senior academics publish in high-ranking journals mentioning in the footnote "excellent research assistance from" often followed by a battery of PhD students. I think that is an abomination. If you cleaned and prepared the data, which is one of the most important parts in the quantitative literature I work in, you should be a co-author, as is the case in the natural sciences.”

Another response from a researcher in Germany offered the following perspective:

“Order of authorship might also be hierarchical, as is common in Germany: the most senior member of the team is usually the lead author even if they have not done anything for the paper at all.”

Cases of honorary authorship have led administrators to divide the scores among the co-authors for promotion purposes. This is not without objections, as some researchers feel that it stifles genuine collaboration.

The issue of who should be the first author can create friction at times, even to the point of it needing to be resolved in court [54]. In interviews with Nobel Laureates that inquired about their name order practices, Zuckerman [49] found that laureates exercise their *noblesse oblige* by giving more credit to less eminent co-workers as their eminence grows.

Hart [20] indicated that authors mentioned various ways in which they listed their names in a co-authored paper, although a vast majority (46.9%) indicated that they listed the names according to the ‘contribution’ of each author. Some of the other methods that can be used include alphabetical order with intent to indicate an equal contribution (15.3%) or without intent to indicate an equal contribution (9.2%). Hart [20] also mentioned cases of ‘helped’ first authorship, where authors of four articles indicated that the first author was in line for tenure and promotion; thus, the co-authors aided to further the individual’s cause by assigning him or her first authorship.

### Distribution of task as a mentor and as a colleague

Research collaboration is categorized into various types, depending on the level of aggregation or model of working relationship. For example, Subramanyam [42] mentioned six different types of collaboration, teacher-pupil collaboration, collaboration among colleagues, supervisor-assistant collaboration, researcher-consultant collaboration, collaboration between and across organizations, and international collaboration. The teacher-pupil relationship is the most common relationship in university-based set-ups where the professor provides guidance or supervision to the student and the student does most of the bench work, hence leading to academic papers. In most cases, both the student and the professor share authorship of these papers. Collaboration among colleagues occurs when authors share the work as colleagues. The teacher-pupil relationship may also be called a ‘mentoring’ relationship or model, and collaboration among colleagues may be called a ‘collegial’ relationship or model [20].

We asked the respondents to indicate if there was a significant difference between the importance of tasks performed in producing a research paper as a mentor and as a colleague. The respondents were asked to rate the tasks (see Table 8) as ‘very important’, ‘important’ and ‘less important’. The statistical results of the Wilcoxon Signed ranks test showed a significant difference between being a mentor and being a colleague in four out of the seven tasks (see Table 8).

**Table 8:** Difference in the tasks performed based on working relationships.

Tasks		Mentor		Colleague		Wilcoxon Signed Rank Test	
	N	Mean	Std. Deviation	Mean	Std. Deviation	Z	Asymp. Sig. (2-tailed)
Writing the paper	580	2.12	.859	2.23	.680	-3.213^a^	0.001*
Collecting the data	580	1.49	.961	1.78	.839	-7.224^a^	0.000*
Analyzing the data	580	1.90	.877	2.08	.754	-5.288^a^	0.000*
Designing the study	580	2.12	.902	2.11	.754	-.395^b^	0.693
Revising the paper	580	2.16	.857	2.12	.707	-1.259^b^	0.208
Reviewing the literature	580	1.61	.860	1.78	.745	-4.860^a^	0.000*
Having the original idea	580	2.06	.965	2.16	.814	-2.399^a^	0.016

* significant p<0.01

a. Based on positive ranks.

b. Based on negative ranks.

These tests signify that, indeed, researchers act differently when it comes to co-authoring with a colleague (collegial) and co-authoring as a mentor.

Two contrasting views of researchers are worth noting here:

“Sometimes, in the past, I’ve been 'invited' to sign my papers with people who were supposed to mentor me but who, in practice, did nothing but sign the paper. This has changed dramatically, and of course, I do not do it with my PhD students.”

“I think there is too much co-authorship going on in economics these days, arguably driven by the goal of getting more citations. I also see an alarming tendency for some people working with their supervisors to get into highly ranked journals and doing nothing or very little of significance on their own. Supervisors have an incentive to do this to attract students who will also do the grunt work but who don't develop their own research agenda or skills for doing original research. Students obviously have the incentive of getting jobs and advancing their careers without being well-rounded scholars”

As mentors, the researchers are mostly either PhD advisors or principal investigators on a research project, having research assistants or postdoctoral scholars working under them. In such cases, the load of the tasks is predominantly on the shoulders of the mentees, whereas mentors provide the guidance. However, depending on the order of authorship or some other pre-arrangements (i.e., equal division of work), the tasks are distributed to co-authors accordingly.

### Preference to associate based on socio-academic parameters

Preference to collaborate with someone due to some kind of similarity or work arrangement is a phenomenon commonly known as ‘assortativity’ or ‘homophily’ [55]. Co-author preference based on nationality, gender, ethnicity, or other factors occur in varying degrees, although they do not usually come to light. For example, Freeman and Huang [56] found that authors with ‘similar ethnicity co-authored more often than expected by their proportion among authors’. Accordingly, we wished to find out whether Economics authors, based on their past records, had shown preferences based on demographic variables, such as same nationality, gender or ethnicity, field of research and professional position.

From our results, we found that, indeed, 38.6% of respondents in this study revealed that they had shown a preference based on nationality at least sometimes (includes ‘most of the time’ and ‘every time’). Regarding gender and ethnicity, 20% of respondents preferred co-authors of the same gender and ethnic background, which is a sizeable percentage. In fact, approximately 15% of respondents revealed that they had shown nationality preference either ‘every time’ or ‘most of the time’. This is, in our opinion, a remarkable figure. We expected that the large percentage of respondents had never shown any preference for any socio-academic parameters. The percentage of non-preference was the highest for ethnicity, gender and nationality and least for ‘friends’. Most researchers showed no preference in terms of nationality, ethnicity or gender.

However, it must be noted that certain preferences may be due to circumstances rather than choices. As one respondent noted:

“Co-authoring with someone of the same gender or ethnic background has never been a factor, but co-authoring with someone from a different gender or background has been important. If you write on gender or cultural topics, it is good to include people from those groups.”

We asked researchers whether they preferred to associate with someone with a professional rank higher than theirs and, if so, to what degree. The survey found that approximately 15% of authors preferred to associate with someone well- known in his/her field either ‘always’ or ‘most of the time’. Researchers, especially those who were new to the research field, preferred to attach themselves to a well-known person in the field. In fact, the very basis for the growth of networks (here a community of researchers) is, in part, preferential attachment [57]. Greater preference was noted for intra- rather than multi-disciplinary work (see Table 9).

**Table 9:** Preference to co-author with other researchers based on socio-academic parameters.

	N	Never	Sometimes	Most of the time	Always	Mean (between 0 to 3)
***Demographics***						
Nationality	580	356	132	74	18	0.58
		(61.4%)	(22.8%)	(12.8%)	(3.1%)	
Gender	580	446	96	28	10	0.31
		(76.9%)	(16.6%)	(4.8%)	(1.7%)	
Ethnicity	580	473	64	33	10	0.28
		(81.6%)	(11.0%)	(5.7%)	(1.7%)	
***Academic***						
Professional rank (higher)	580	251	242	76	11	0.74
		(43.3%)	(41.7%)	(13.1%)	(1.9%)	
Professional rank (equal)	580	266	236	71	7	0.69
		(45.9%)	(40.7%)	(12.2%)	(1.2%)	
My juniors/students	580	215	290	70	5	0.77
		(37.1%)	(50.0%)	(12.1%)	(.9%)	
Department	580	180	275	113	12	0.93
		(31.0%)	(47.4%)	(19.5%)	(2.1%)	
***Social***						
Friends	580	176	263	116	25	0.98
		(30.3%)	(45.3%)	(20.0%)	(4.3%)	

When asked about their preference for collaboration based on equal professional position, again, a high percentage showed this preference. Over 15% revealed that they preferred to work with their juniors/students ‘always’ or ‘most of the time’. These results reveal that authors do indeed have strong preferences (‘always’ and ‘most of the time’), albeit with a smaller overall percentage, when co-authoring a paper. Researchers’ preference to work with someone from the same department is logical, as geographical proximity makes it more conducive for researchers to carry out research together. Over 21.5% of the researchers in our study mentioned that they prefer a department colleague most or all of the time.

Preference to associate due to friendship is comparatively more common compared to preference due to the demographic profile of a researcher. These preferences (i.e., friendship with someone well known in the field) may be even required to flourish in the field. Researchers strategize in different ways to improve their academic standing; thus, showing these associations makes sense, too. Friendship ranked the highest in terms of preference (mean 0.98). Friendship could be an important catalyst in their later decision to collaborate on a paper. After all, the co-authorship decision occurs purely in the social domain–researchers choose who they want to co-author paper with.

## Conclusions

Our study surveyed 580 researchers worldwide to understand Economics authors’ perceptions of research authorship and collaboration. The survey revealed that almost all respondents had co-authored a paper at least at one time in their academic life, with 75% of the respondents co-authoring a majority (two-thirds or more) of their papers. Significant differences in the proportion of co-authored papers was observed among respondents based on age, gender and the number of years they had spent in their present institution. Concerning the benefits and motivation for co-authorship, the respondents indicated the improvement in the quality of the research paper followed by mutual gain of expertise and division of labor as the biggest benefits of co-authorship. Economics authors are known to follow an alphabetical order of authorship. However, our study found that a considerable percentage (34.5%) of researchers co-authored the papers based on significant contribution of work. With respect to writing the paper, significant differences were found in the distribution of tasks depending on the working relationship between the authors, whether it was colleague-colleague or mentor-mentee. Lastly, it was revealed that researchers did have preferences, to varying degrees, regarding who to associate with based on various socio-academic parameters.

## Supporting Information

S1 Questionnaire Contains questionnaire used for the online survey. (PDF)

S1 Data Contains data used for analysis. (XLSX)
