# Supplementary material for: Perceptions of Scholars in the Field of Economics on Co-Authorship Associations: Evidence from an International Survey
Source: PLoS One. 2016 Jun 20;11(6):e0157633. doi: 10.1371/journal.pone.0157633 (PMC4913925; doi:10.1371/journal.pone.0157633)
Supplement: S1 Questionnaire — (PDF) [file pone.0157633.s001.pdf]

Edit this form

Survey on the Perceptions of co-authorship associations among Academic Economists

Dear Professor

I am carrying out a new world-wide survey (2015) to understand the perceptions of Academic Economists on Co-authorship associations. ('Co-authorship' means writing research paper/s with two or more authors).The survey takes about 10 to 15 minutes of your valuable time. No personal details that would identify any individual respondent, will be ever disclosed. Results will be reported only in the aggregate.

I thank you for giving your valuable time for this survey.

* Required

Age *

Your Age bracket (in years)

- less than 35
- 35-45
- 46-55
- 56 and above

Nationality *

Which country's nationality do you hold (i.e. Japan, Malaysia, Indonesia, Germany, USA, etc.)

Gender *

- Male
- Female

Marital Status *

- Married
- Single
- Other:

Your highest qualification *

- PhD
- Masters
- Other:

Where do you work? *

- University
- College
- Research Institute
- Other: ______

In which Country is your University (or workplace) located? *

______

Your University/College/Research Institute Department *

In case you work at an academic institution (University or College or Research Institute), kindly mention your department (i.e. Department of Economics). Please mention 'n/a' in case you do not work within a department.

______

Number of years of service at your current institution *

- Less than 1 year
- 1 - 5 years
- 6 -10 years
- More than 10 years

Professional Position *

- Professor
- Associate Professor
- Assistant Professor
- Senior Lecturer
- Lecturer
- Other: ______

Approximately what proportion of peer-reviewed research papers have you co-authored with other researchers? *

Papers that are co-authored by two or more researchers, in which you have been a co-author too

- All papers
- Almost all papers
- About two-third
- About half
- About one-third
- Very few
- None. All have been solo written by me

Benefits and Motivations of Collaboration

From your perception, what do you think are the benefits and motivations of collaborating with other researchers (co-authorship) when writing a research paper? *

	Not Important	Slightly Important	Important	Very Important
Improvement in the quality of Research paper	○	○	○	○
Increase in the no. of publications thereby helping in promotion or tenure	○	○	○	○
Division of labour (work gets divided among co-authors thereby reducing individual author work load)	○	○	○	○
Mutual gain of expertise among co-authors (i.e. learning new tools or techniques from your co-author/s)	○	○	○	○
Opportunity to know a colleague	○	○	○	○
Mentor a junior colleague	○	○	○	○
Be mentored by a senior colleague	○	○	○	○
Opportunity to work with co-authors from International institutions	○	○	○	○
Reduction in research costs because of sharing of resources	○	○	○	○
Opportunity to work on multi-disciplinary areas	○	○	○	○
Opportunity to be part of a large international or cross-country research (which normally involves researchers from several countries)	○	○	○	○
Establishing further networks (i.e. knowing co-authors of your co-authors)	○	○	○	○

Models of Working Relationship with co-authors as a

'Mentor' and as a 'Colleague'

As a MENTOR, kindly mention the importance of tasks of producing a Research Paper *

'Mentoring Model' working relationship is when you are co-authoring a paper with one or more junior researchers/students

	Not Important	Slightly Important	Important	Very Important
Writing the paper	○	○	○	○
Collecting the data	○	○	○	○
Analyzing the data	○	○	○	○
Designing the study	○	○	○	○
Revising the paper	○	○	○	○
Reviewing the literature	○	○	○	○
Having the original idea	○	○	○	○

As a COLLEAGUE, kindly mention the importance of tasks of producing a Research Paper *

'Collegial Model' Working relationship when you are co-authoring a paper with one or more of your Colleagues

	Not Important	Slightly Important	Important	Very Important
Writing the paper	○	○	○	○
Collecting the data	○	○	○	○
Analyzing the data	○	○	○	○
Designing the study	○	○	○	○
Revising the paper	○	○	○	○
Reviewing the literature	○	○	○	○
Having the original idea	○	○	○	○

Order of Authorship

How do the names of authors generally appear (order of authorship) on a Research Paper co-authored by you? *

	In none of my papers	Very few of my papers	In about one-third of my papers	In about half of my paper	In about two-third of my papers	Almost all my papers	In all my papers
In order of significant Contribution	○	○	○	○	○	○	○

Alphabetically, indicating an equal contribution by each author	○	○	○	○	○	○	○
Alphabetically, but no intent to indicate significant contribution	○	○	○	○	○	○	○

Assortativity (Preference to associate with a co-author)

Looking at your past record, have you preferred co-authoring a paper with someone due to one or more of the following socio-academic parameters? *

Kindly note that option 'Never' stands for 'Never has been a preference factor' or 'it just does not matter for me' .

	Never	Sometimes	Most of the time	Always
Those with the same nationality	○	○	○	○
Those with the same gender	○	○	○	○
Those with the same ethnicity	○	○	○	○
Those with professional rank higher than me	○	○	○	○
Those with equal academic rank	○	○	○	○
My juniors/students	○	○	○	○
Those from my University/Institution department	○	○	○	○
My friends	○	○	○	○

Kindly mention any other points or ideas about Co-authorship you would like to share with us.

By filling up and sending this online survey form as a respondent, I have consented to voluntarily participate in the study.

Submit

Never submit passwords through Google Forms.

Powered by

This form was created inside of University of Malaya.

Report Abuse - Terms of Service - Additional Terms
